# The tissue-specific chromatin accessibility landscape of *Papaver somniferum*

**DOI:** 10.3389/fgene.2023.1136736

**Published:** 2023-03-15

**Authors:** Yanyan Jia, Yu Xu, Bo Wang, Li Guo, Mengyao Guo, Xiaofei Che, Kai Ye

**Affiliations:** ^1^ School of Automation Science and Engineering, Faculty of Electronic and Information Engineering, Xi’an Jiaotong University, Xi’an, Shaanxi, China; ^2^ School of Life Science and Technology, Xi’an Jiaotong University, Xi’an, Shaanxi, China; ^3^ MOE Key Lab for Intelligent Networks & Networks Security, Faculty of Electronic and Information Engineering, Xi’an Jiaotong University, Xi’an, Shaanxi, China; ^4^ Genome Institute, the First Affiliated Hospital of Xi’an Jiaotong University, Xi’an, Shaanxi, China; ^5^ Faculty of Science, Leiden University, Leiden, Netherlands

**Keywords:** BIA biosynthesis, ATAC-seq, tissue-specific, regulatory mechanisms, Papaver somniferum (poppy)

## 1 Introduction

Opium poppy (*Papaver somniferum*) is a flowering plant in the Papaveraceae family that has been valued for its ornamental and significant medicinal properties for thousands of years (Norn et al., 2005; Singh et al., 2019; Lal, 2022). It produces several pharmacologically active benzylisoquinoline alkaloids (BIAs), such as morphine, codeine, thebaine, and noscapine, which make opium poppy the only natural source of commercial opiates worldwide and potentially play roles in plant defense against biotic and abiotic challenges (Singh et al., 2019). Developmental regulation of BIA biosynthesis facilitates organ- and tissue-specific accumulation of major alkaloids. Primary alkaloids mainly accumulate in the stems and capsules of mature plants (Facchini and De Luca, 1995; Hagel and Facchini, 2013; Beaudoin and Facchini, 2014). However, the regulatory mechanisms behind tissue-specific production and enrichment of natural products in opium poppy are largely unknown (Yucebilgili Kurtoglu and Unver, 2021). Only a few studies have investigated the implications of transcription factors (TFs) in BIA biosynthesis in opium poppy (Mishra et al., 2013; Kakeshpour et al., 2015; Agarwal et al., 2016; Yucebilgili Kurtoglu and Unver, 2021). Previously, we reported that a majority of genes that encode enzymes for metabolic pathways of BIAs are not only clustered in the opium poppy genome but also co-expressed in stem, capsule and root tissues (Guo et al., 2018; Yang et al., 2021). The mechanism by which co-expression of BIA genes occurs selectively in some tissues but not in others is intriguing and unknown. Therefore, we aimed to sequence and study the epigenome and transcriptome of distinct opium poppy tissues to uncover the tissue-specific regulatory mechanisms of general plant development, particularly BIA production.

Accessible chromatins regions (ACRs) located at promoters, enhancers, and other gene regulatory regions allow TFs to bind, which is crucial for transcriptional regulation during a wide range of developmental and metabolic processes (Thurman et al., 2012; Yocca and Edger, 2022). At present, transposase accessible chromatin sequencing (ATAC-seq) is an emerging technology for detecting the highly opened ACRs and subsequently, identifying TF-binding sites within these regions. ATAC-seq has been widely employed in recent years for large-scale identification of open chromatin in mammals, fungi and plants as a quicker and more efficient approach (Lu et al., 2017; Maher et al., 2018; Sijacic et al., 2018; Klemm et al., 2019; Pawlak et al., 2019; Chen et al., 2021), However, no study has yet examined *P. somniferum*. Here, we conducted a comprehensive tissue-specific assay for ATAC-seq and transcriptome sequencing (RNA-seq) analysis of six different tissues in the opium poppy to dissect the epigenetic and transcriptional regulatory mechanisms for tissue-specific BIA metabolism. In this study, we discovered that HB6 is a key transcription factor that regulates the expression of the BIA gene cluster. This first tissue-specific chromatin accessibility landscape of *P. somniferum* provides an important resource for functional epigenetic analysis and future research aimed at characterizing or using gene regulatory elements for breeding poppy varieties with high BIA content.

## 2 Materials and methods

### 2.1 Plant materials and growth conditions

Opium poppy cultivar HN1 seeds were sowed in a soil mix containing potting mix, vermiculite, and sand at a 2:1:1 ratio. The seeds were incubated in plant growth chambers under a 16-h light and 8-h dark cycle at 22°C and 60% humidity. Six different tissues, including the leaves, stems (2–4 cm below the capsule), capsules, petals, tap roots, and fine roots of opium poppy plants were harvested 1-day post-anthesis, frozen immediately in liquid nitrogen and stored in a freezer at −80°C. Each sample has three biological replicates. Half of the above materials were used for RNA-seq and the other half for ATAC-seq.

### 2.2 Nuclei isolation and ATAC sequencing

To understand the regulatory dynamic epigenomic mechanisms that underpin the distinctive tissues, we performed ATAC-seq for six different tissues of *P. somniferum*. A quantity of 1–3 g from the six different fleshy tissues was ground into powder in liquid nitrogen and then the nuclei were isolated as described previously (Bajic et al., 2018). The isolated nuclei were used to build the library with Novoprotein Chromatin Profile Kit (Novoprotein, #N248) following the company’s recommended protocols: The isolated nuclei were immediately resuspended in the Tn5 transposase reaction mix. The transposition reaction was incubated at 37 C for 30 min. Equimolar Adapter 1 and Adapter 2 were added after transposition and then PCR was performed to amplify the library. After PCR, the libraries were purified with the AMPure beads and library quality was assessed using Qubit. The library preparations were sequenced after cluster generation on an Illumina Hiseq platform and 150 bp paired-end reads were generated. All ATAC-seq processing was performed by Novogene Technology Inc. (Tianjin, China).

### 2.3 RNA extraction and sequencing

To understand the tissue-specific transcriptional process in opium poppy, we performed RNA-seq for six different tissues of *P. somniferum*. The plant materials used for RNA-seq analysis were the same as those used for ATAC-seq. Total RNA was extracted from six different tissues using Trizol reagent (Ambion, #15596018) according to the manufacturer’s instructions. RNA integrity was determined using regular agarose gel electrophoresis, Nanodrop (ThermoFisher Scientific, United States), and Agilent 2100 Bioanalyzer (Agilent Technologies, United States). RNA sample of high quality (OD260/280 within the range [1.8, 2.2], OD260/230 ≥ 2.0, RIN ≥8) was used to construct the sequencing library. Library construction and sequencing were performed by Novogene Technology Inc. (Tianjin, China) with Hiseq platform (Illumina Inc, United States) using the paired-end sequencing strategy (150 bp for each end). All the tissues were subjected to three biological replicates.

### 2.4 RNA sequencing data analysis

The purpose of this analysis was to quantify genome-wide gene expression levels and identify transcriptional diversity in *P. somniferum*. Pair-end RNA-seq reads were first assessed for quality by FastQC v0.10.1 (Andrews, 2010). Trimmomatic was used to remove sequence adapters and reads of low quality (Phred Q < 20) (Bolger et al., 2014). High-quality and clean RNA-seq reads were mapped to the reference genome of *P. somniferum* HN1 (Yang et al., 2021) using bowtie2/2.3.5 (Langmead and Salzberg, 2012). Mapped reads were filtered using Samtools to retain only those that had a mapping quality score of 10 or higher (Samtools “view” command with option “-q 10” to set mapping quality cutoff) (Li et al., 2009). Filtered reads were used to construct transcriptome by Cufflinks/2.2.1 (Trapnell et al., 2012). Dimension reduction (PCA analysis) was performed using FactoMineR (Lê et al., 2008).

### 2.5 ATAC sequencing data analysis

The purpose of this analysis was to identify and map genome-wide cis-regulatory elements involved in transcriptional regulation in *P. somniferum*. The cleaned reads were mapped to the *P. somniferum* genome using BWA (Li and Durbin, 2009) software with “mem” parameters (Yang et al., 2021). Mapped reads in sam format were converted to bam format and sorted using Samtools v1.9 and Sambamba ‘markdup’ command was then used to remove PCR duplicates (Li et al., 2009; Tarasov et al., 2015). Reads with higher mapping quality scores (MAPQ ≥10) were employed to perform downstream analysis.

ATAC-seq peak calling was conducted using Genrich with default recommended parameters (v0.6, “-j -r -v”, available at https://github.com/jsh58/Genrich). Intervene was used to intersect different samples to obtain tissue-specific peaks and overlapped peaks (Khan and Mathelier, 2017). The number of reads of each genome region was counted using the ‘multiBamSummary’ script in deepTools v2.0 and PCA were performed with FactoMineR (Lê et al., 2008; Ramírez et al., 2016).

For each ATAC-seq data set, the peaks were assigned to genes using the R/Bioconductor package ChIPseeker (Yu et al., 2015). This program assigns each peak to the closest TSS, whether promoter, downstream, distal intergenic, intron, exon, 5′UTR, or 3′UTR, and reports the distance from the peak center to the TSS based on the genome annotations (Yang et al., 2021). The TF motif enrichment analysis on ATAC-seq data was performed using the ‘findMotifsGenome.pl’ function of HOMER package (Heinz et al., 2010). TF binding sites were performed using the ‘annotatePeaks.pl’ function of HOMER package (Heinz et al., 2010).

### 2.6 Visualization

In order to better visualize the results of the data analysis, we performed a variety of visualization tools. The filtered, sorted and scaled bam files were converted to the bigwig format for visualization using the BAMscale with default parameters (Pongor et al., 2020). Genome browser images were created using the Integrative Genomics Viewer (IGV) v2.8.10 (Thorvaldsdóttir et al., 2013) and bigwig files were processed as described above.

Motif binding regions were visualized using ggmsa (v0.0.6, available at https://cran.r-project.org/web/packages/ggmsa/index.html). A Venn diagram for ATAC-seq and RNA-seq samples was generated using R package Venn (v1.9, available at https://cran.r-project.org/web/packages/venn/). The distribution of ATAC-seq peaks was visualized with Circos v0.69-8 (Krzywinski et al., 2009). A Heatmap was generated using pheatmap package and bar and pie plots were all created using ggplot2 package (Wickham, 2016; Kolde, 2019).

### 2.7 Code access

The methods related program source code has been submitted to GitHub (https://github.com/) under URL https://github.com/StuYuXu/Chromatin-accessibility-landscape-of-Papaver-somniferum.

## 3 Results

### 3.1 Identification of accessible chromatin regions in *Papaver somniferum* by ATAC-seq

**FIGURE 1:**
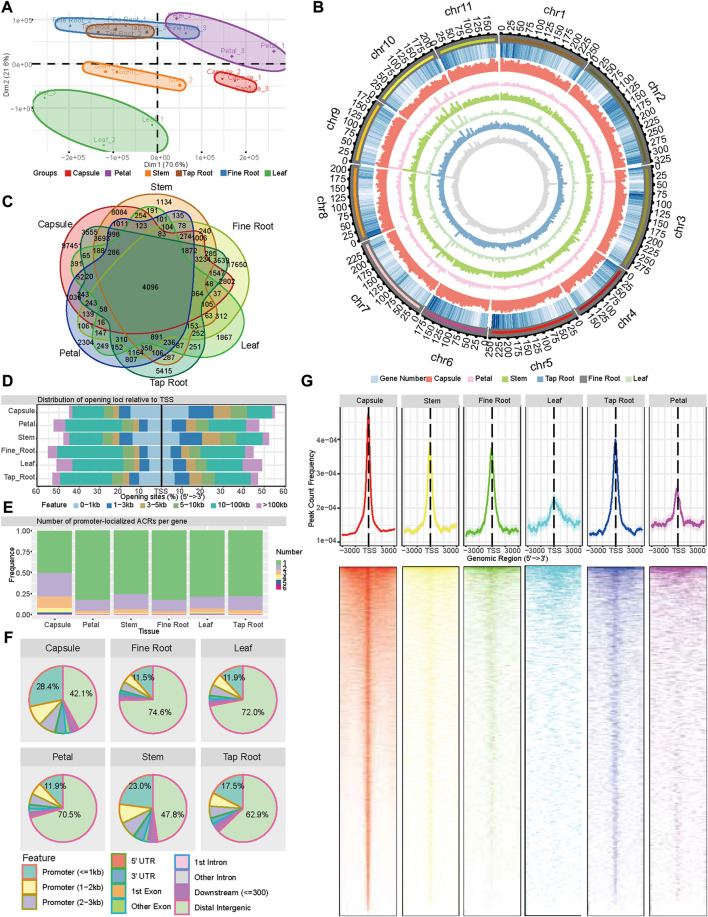
Landscape of accessible chromatin regions across six tissues (capsule, leaf, petal, stem, tap root, and fine root) of *P. somniferum*. **(A)** Graphical representation of PCA of opium poppy ATAC-seq data between all the biological replicates across the six tissues based on ACR. **(B)** Genome-wide distribution of ACRs along 11 opium poppy chromosomes. From outside to inside, the circles represent the chromosome, gene density, ACRs abundance in the capsule, ACRs abundance in the petal, ACRs abundance in the stem, ACRs abundance in the leaf, ACRs abundance in the tap root, and ACRs abundance in the fine root. **(C)** Venn diagram displaying the number of common and unique ACRs among the six distinctive tissues. **(D)** Distribution of ACRs relative to genes in each tissue. **(E)** Number of promoter-localized ACRs per gene in each tissue. **(F)** Genomic annotation and distribution of ACRs in the six tissues. **(G)** Distribution of promoter-localized ACRs in the six tissues.

We performed chromatin accessibility profiling using ATAC-seq for six different tissues of *P. somniferum* HN1 variety, including the leaves, petals, stems, capsules, tap roots, and fine roots. The tissues were all harvested on the first day of anthesis. The ATAC-seq libraries of three biological replicates for each tissue were sequenced using Illumina paired-end sequencing, yielding a total of 890 million clean reads that were mapped to the reference genome of *P. somniferum* variety HN1 (Yang et al., 2021) (Supplementary Table S1). Based on accessible chromatin region profiles, the principal component analysis (PCA) of the ATAC-seq data showed that the three biological replicates within each tissue were highly correlated and roughly separated into tissue-specific clusters (Figure 1A), with the exception that the clusters of tap root and fine root intermingled with each other (Figure 1A).

To better understand the tissue-specific transcriptional regulation in opium poppy, we also performed RNA-seq from the same six tissues of which epigenomes were assessed. Based on gene expression profiles, the PCA showed a clean separation of various tissue types (Supplementary Figure 1A), except for tap roots and fine roots, reflecting a correlation of the two tissues in both chromatin accessibility and gene transcription. With a threshold of transcripts per million (TPM) larger than 1, the number of expressed genes per tissue ranged from 24,537 (petals) to 31,768 (capsules), representing approximately 44%–57% of the total genes of *P. somniferum*, respectively (Supplementary Figure 1B). Comparison of the expressed genes among the tissues shows that approximately 36% of the genes (19,682) were expressed in all the six tissues (Supplementary Figure 1B), with each tissue having various numbers (0.5%–1.2%) of uniquely expressed genes. The capsules contained the most tissue-specifically expressed genes (663), out of the six tissues studied (Supplementary Figure 1B). The fact that the capsule has the most expressed and unique genes of any tissue underlines the transcriptional and physiological hyperactivity in this tissue.

Then, in order to better understand the regulatory dynamic mechanisms underpin the distinctive tissues, we identified a series of ATAC-seq peaks representing ACRs using Genrich software (https://github.com/jsh58/Genrich) for each tissue. As a result, 133,374, 18,914, 31,974, 11,525, 35,877, and 43,565 ACRs were identified in the capsules, petals, stems, leaves, tap roots, and, fine roots, respectively (Figures 1B,C; Supplementary Table S2). The comparison of ACRs in the different tissues showed that 4,096 ACRs were shared by all the tissues, with the capsule having the most tissue-specific ACRs (Figure 1C). Examining the location of ACRs relative to genes showed that in all the tissues, except the capsule (46.1%) and stem (40.3%), approximately 20%–30% ACRs were located within 3 kb regions of the transcription start site (TSS) (Figure 1D), while 42.1%–74.6% of ACRs were located in distant intergenic regions. In comparison to other tissues, the capsule (28.4%) and stem (23.0%) contained more ACRs within 1 kb promoter regions and fewer ACRs in distal intergenic regions (Figure 1F). As for the genes with a detected promoter region of the ACRs across the six tissues (region from −3,000 to 3,000 bp relative to the TSSs of genes, pACRs), 80%, 12%–18%, and 3%–5% had a single ACR, two ACRs, and three ACRs, respectively, except for the capsule, which had 50%, 28%, and 14%, respectively (Figure 1E). The peak of the promoter-localized ACR was located around TSS for all the six tissues, with 61.47%, 56.95%, 58.87%, 55.8%, 52%, and 51.74% within the 1 kb of TSS in the capsule, stem, tap root, fine root, leaf, and petal, respectively, demonstrating a more open chromatin state around TSS than the rest of promoter regions (Figure 1G). Taken together, the ATAC-seq detected a large amount of ACRs with a distinct distribution associated with six tissues, reflecting a common and distinct state of open chromatins among these tissues.

### 3.2 Tissue-specific chromatin accessibility and transcription of BIA gene cluster

**FIGURE 2:**
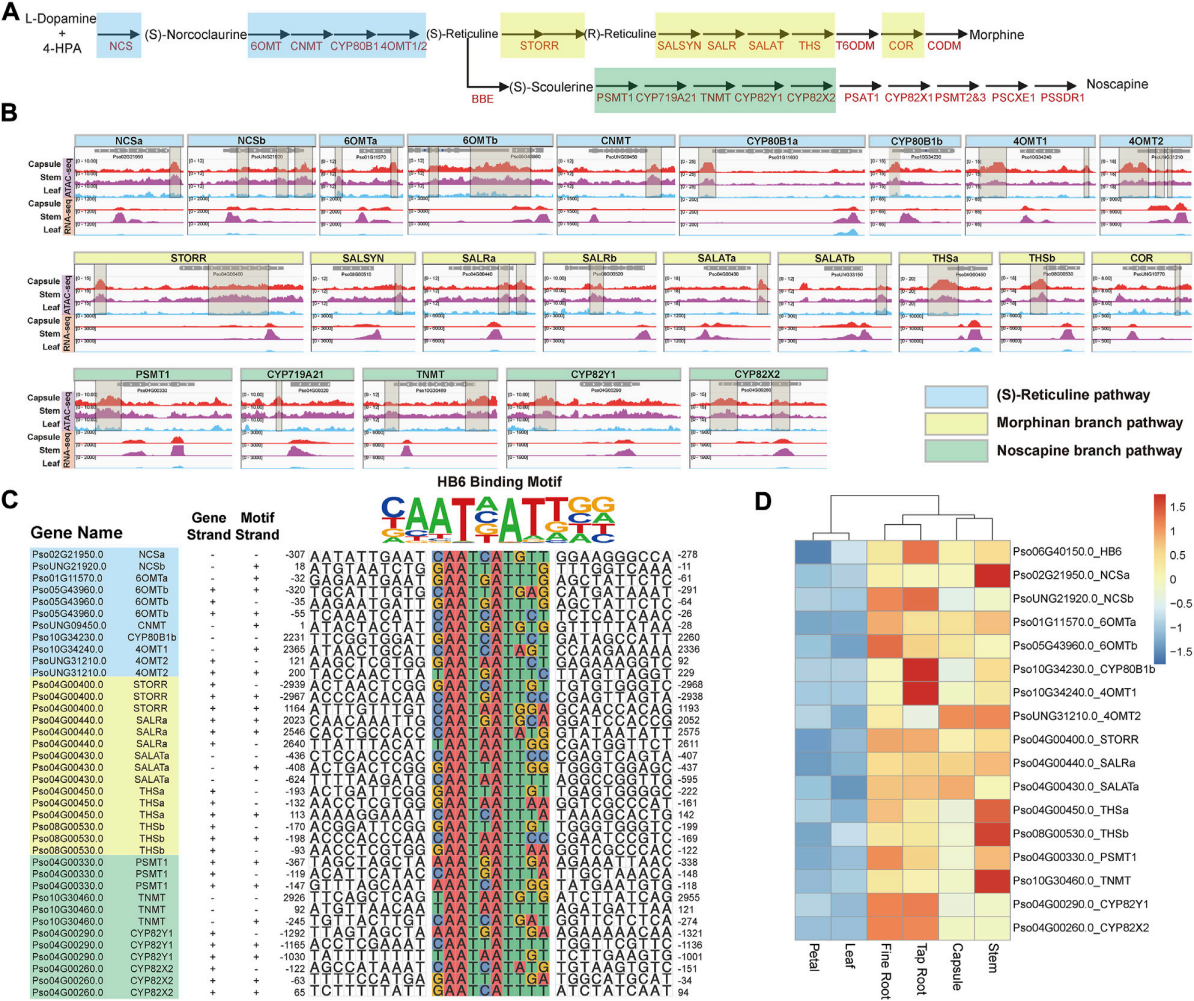
Coordinate regulation of BIAs metabolic pathway genes. **(A)** Schematic representation of (*S*)-reticuline pathway and noscapine and morphinan branch pathways. **(B)** Visualization of ACRs from −3,000 to 3,000 bp relative to the TSS of these isoquinoline metabolic pathway genes. The brown transparent boxes show ACRs. **(C)** The predicted cis-regulatory element within the region from −3,000 to 3,000 bp relative to the TSS of (*S*)-reticuline, noscapine, and morphinan metabolic pathway genes. **(D)** The expression patterns of HB6 as well as its predicted regulated genes.

*Papaver somniferum* production of pharmaceutically valuable BIAs, such as morphine, codeine, thebaine, and noscapine is a distinguishing characteristic (Beaudoin and Facchini, 2014). The genes encoding the BIA biosynthetic pathway are well characterized and partly of these are organized in an outstanding gene cluster on the *P. somniferum* genome (Figure 2A) (Guo et al., 2018; Yang et al., 2021). The fact that these BIA genes are transcriptionally co-regulated in a tissue-specific (capsule, root, and stem) manner (Supplementary Figure S2) raises the question of how this is achieved epigenetically and what regulatory elements are involved. Using enrichment analysis of DNA *cis*-regulatory elements in ATAC-seq data, we detected 43 capsule-, stem-, and root-specific pACRs 3 kb upstream of most BIA biosynthesis genes that encode the (*S*)-reticuline pathway (*NCS*, *6OMT*, *CNMT*, *CYP80B1*, and *4OMT*) and sequentially convert L-dopamine and 4-HPA into the (*S*)-reticuline, morphinan branch (*STORR, SALSYN, SALR, SALAT, THS,* and *COR*), and noscapine branch (*PSMT1, CYP719A21, TNMT, CYP82Y1*, and *CYP82X2*) pathways, which is in accordance with their capsule-specific, root-specific and stem-specific gene expression (Figure 2B; Supplementary Table S3). In contrast, we did not observe any ACRs of these genes in the non-BIA producing tissues, such as the leaf and petal, where they are lowly expressed (Figure 2B). These findings provide evidence that the chromatin becomes accessible to certain unknown transcriptional regulators (e.g., TFs) in specific tissues allowing them to activate the transcription of many of these BIA genes.

Subsequently, we identified potential TFBS from the 43 pACRs associated with BIA genes through motif enrichment analysis (Figure 2B; Supplementary Figure S3; Supplementary Table S3). The HB-HD-ZIP family and the WRKY family recognition motifs were significantly enriched among these pACRs, which is consistent with previous studies on the function of WRKY family proteins in the regulation of BIA biosynthesis in California poppy (*Eschscholzia californica*) (Yamada et al., 2021). Nevertheless, combining the results of motif enrichment and RNA-seq analysis, *HB6*, a HB-HD-ZIP transcription factor, emerged as a key regulator in BIA biosynthesis in *P. somniferum*. Specifically, *Pso06G40150.0*, which encodes a *P. somniferum* homolog of *Arabidopsis HB6*, might regulate 19 BIA biosynthetic genes including eight in the (*S*)-reticuline pathway, five in the morphinan branch pathway, four in the noscapine branch pathway, one (*P6H*) in the sanguinarine branch pathway, and one (*7OMT*) in the laudanine branch pathway (Figure 2C; Supplementary Figure S4). However, *HB6* was only highly co-expressed with 17 genes with significant *p* values calculated by 18 samples (average correlation coefficient >0.818) (Figure 2D; Supplementary Table S4). For instance, *Pso04G00400.0* (*STORR*) is a pivotal gene in the morphine branch pathway, which contained *HB6* binding motif within its promoter region. This motif was significantly opened in the capsule, stem, fine root, and tap root, but nearly closed in the petal and leaf, which was consistent with its lower expression level in the petal and leaf (Winzer et al., 2015) (Figures 2B,C; Supplementary Figure S5). The remaining 16 genes yielded similar findings (Figures 2B,C). In summary, these results suggested that *Pso06G40150.0* might be a key transcription factor in regulating the expression of the BIA gene cluster.

Taken together, our findings suggest that several major genes involved in the BIAs metabolic pathway may be regulated coordinately by the same transcription factor.

## 4 Conclusion

*Papaver somniferum*, one of the most important medicinal plants in the world, has been widely used in clinical medicine for thousands of years due to its unique ability to produce a variety of active alkaloids, including noscapine, morphine, and codeine, all of which have potential pharmacological activity in relieving pain, cough, muscle relaxation, anticancer, etc. (Yamada et al., 2021). However, the regulatory mechanisms governing its development and tissue-specific product synthesis remain unclear. These research status limit full utilization and breeding improvement of *P. somniferum.*

In this study, we constructed the first *cis*-regulatory elements landscapes from six distinctive tissues (i.e., capsule, stem, fine root, tap root, leaf, and petal) and provided paired transcriptomic data in *P. somniferum*. Our approach, which combines chromatin accessibility profiling with transcriptome profiling, is practicable and precise for identifying *cis*-regulatory elements and building regulatory networks. Our data atlas provides a valuable resource for the study of epigenetic mechanisms underlying plant development and secondary metabolism.

Future research should focus on following two aspects of research. First, RNA-seq and ATAC-seq data analyses predicted that HB6 serves as a key TF to co-regulate 17 BIA biosynthetic genes. However, it is not yet clear how HB6 regulates these 17 genes. Therefore, future studies will have to elucidate the molecular mechanism underlying HB6 action. Second, our study found that two independent biosynthetic genes were co-localized to form a big gene cluster as well as co-expressed and co-regulated. However, the evolution of this process is still unclear, especially how this gene cluster is formed and these regulatory elements evolved. We believe that, in the future, our valuable resource will help us solve these puzzles.

## Data Availability

The datasets presented in this study can be found in online repositories. The names of the repository/repositories and accession number(s) can be found below: https://www.ncbi.nlm.nih.gov/bioproject?term=PRJNA746779.
