# Supplementary figures and images for: The tissue-specific chromatin accessibility landscape of Papaver somniferum
Source: Front Genet. 2023 Mar 15;14:1136736. doi: 10.3389/fgene.2023.1136736 (PMC10050356; doi:10.3389/fgene.2023.1136736)

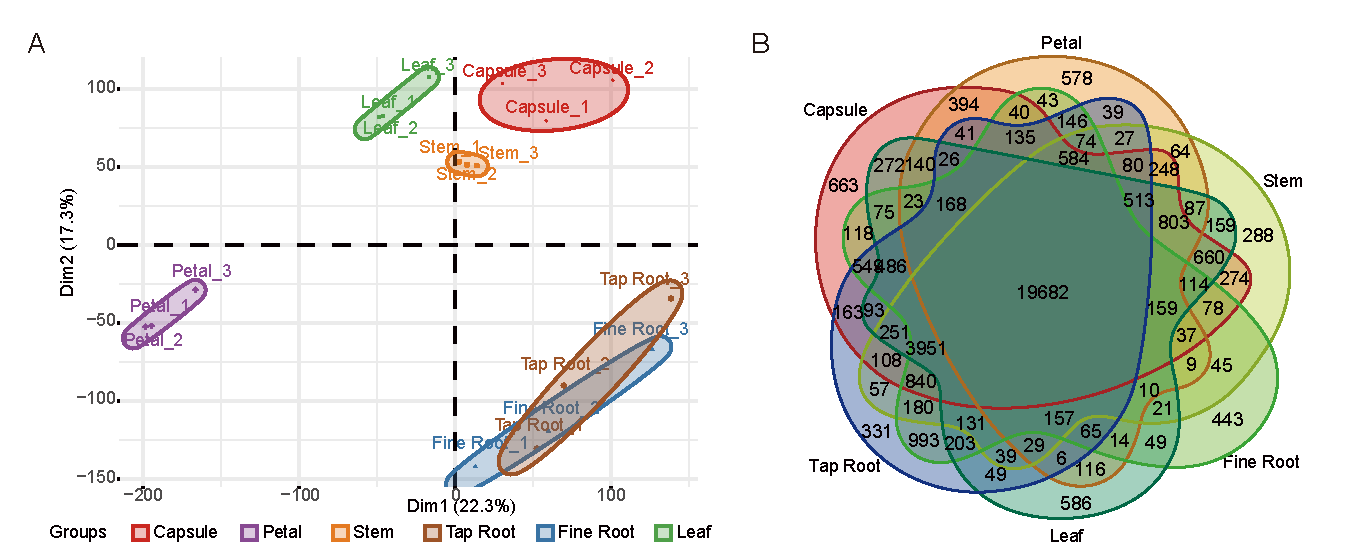

Supplement: Supplementary file 1 [file DataSheet1.ZIP › supplementary material/Supplementary Figure 1.tif]

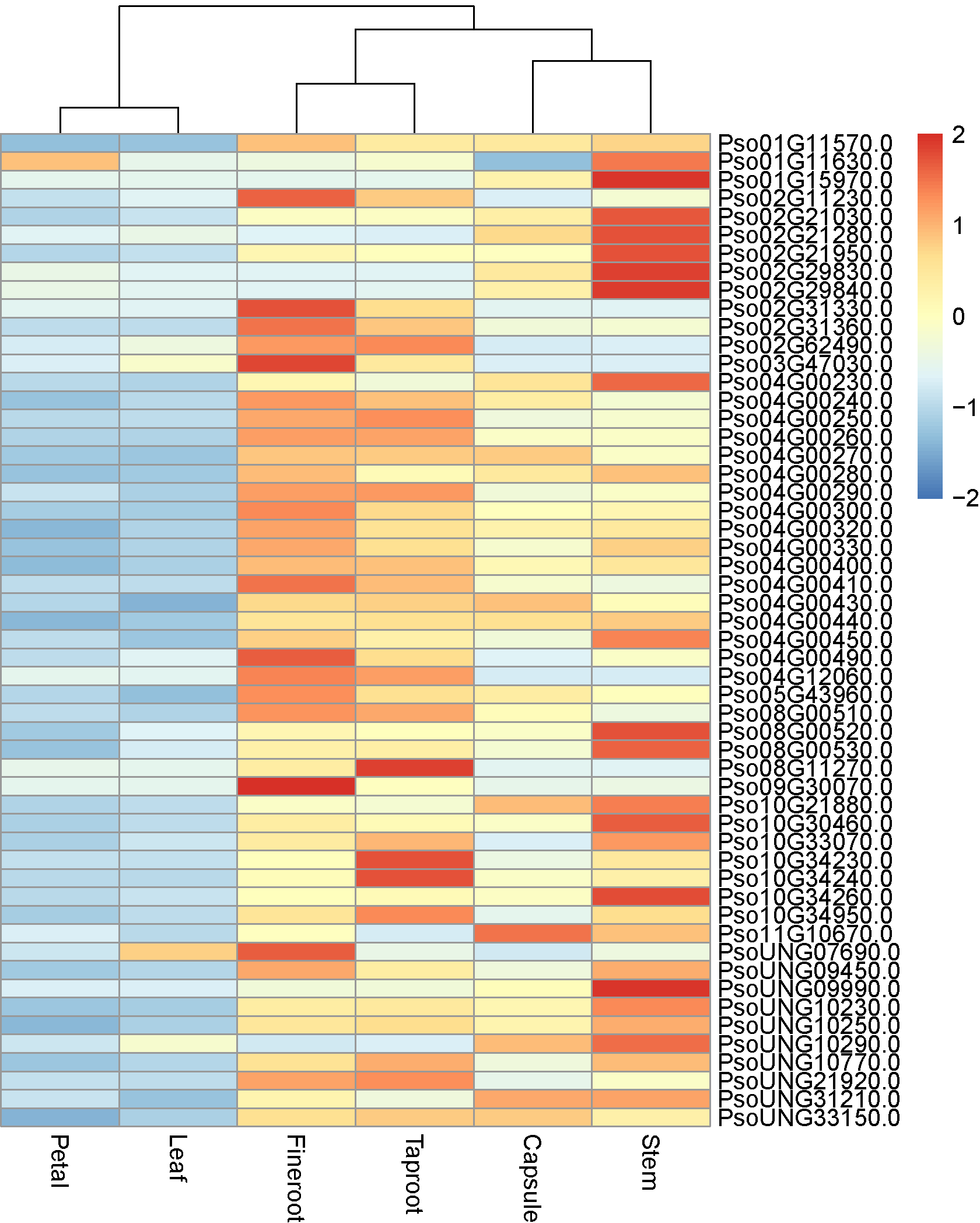

Supplement: Supplementary file 1 [file DataSheet1.ZIP › supplementary material/Supplementary Figure 2.tif]

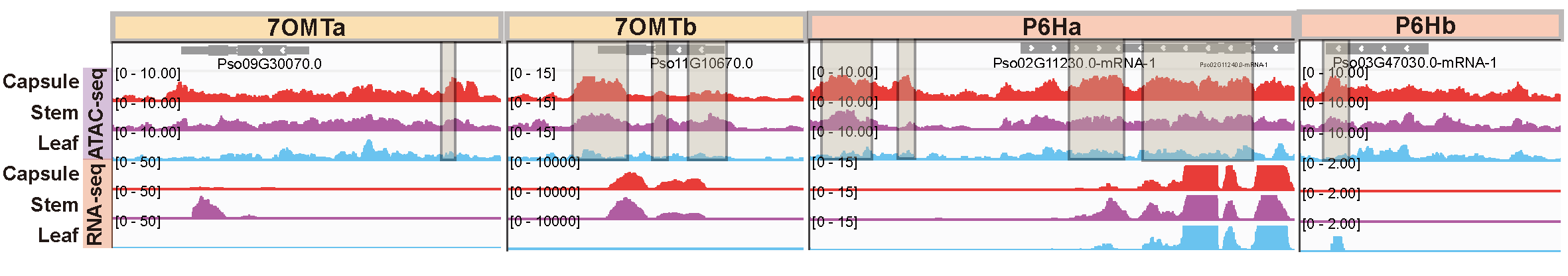

Supplement: Supplementary file 1 [file DataSheet1.ZIP › supplementary material/Supplementary Figure 3.tif]

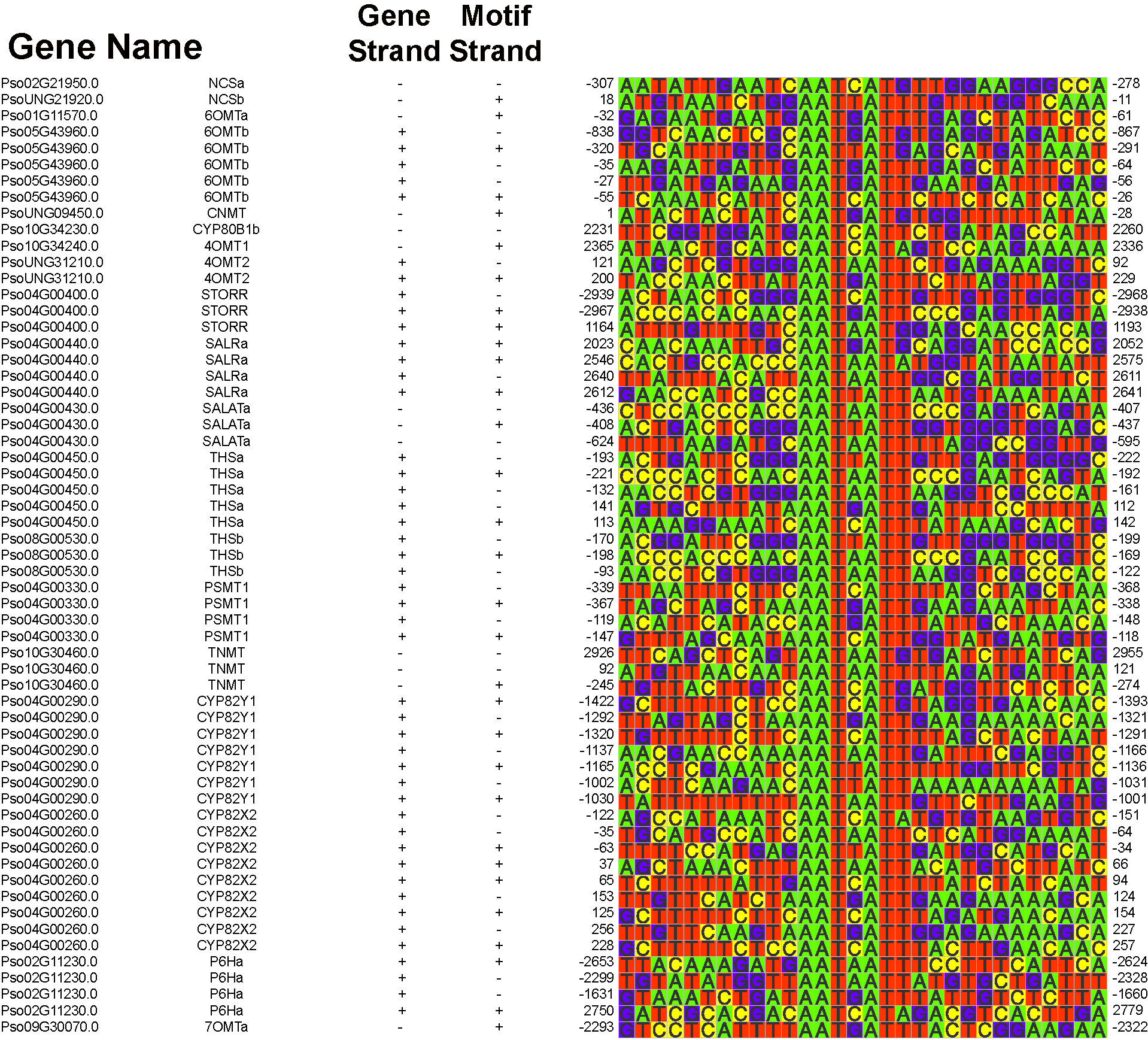

Supplement: Supplementary file 1 [file DataSheet1.ZIP › supplementary material/Supplementary Figure 4.tif]

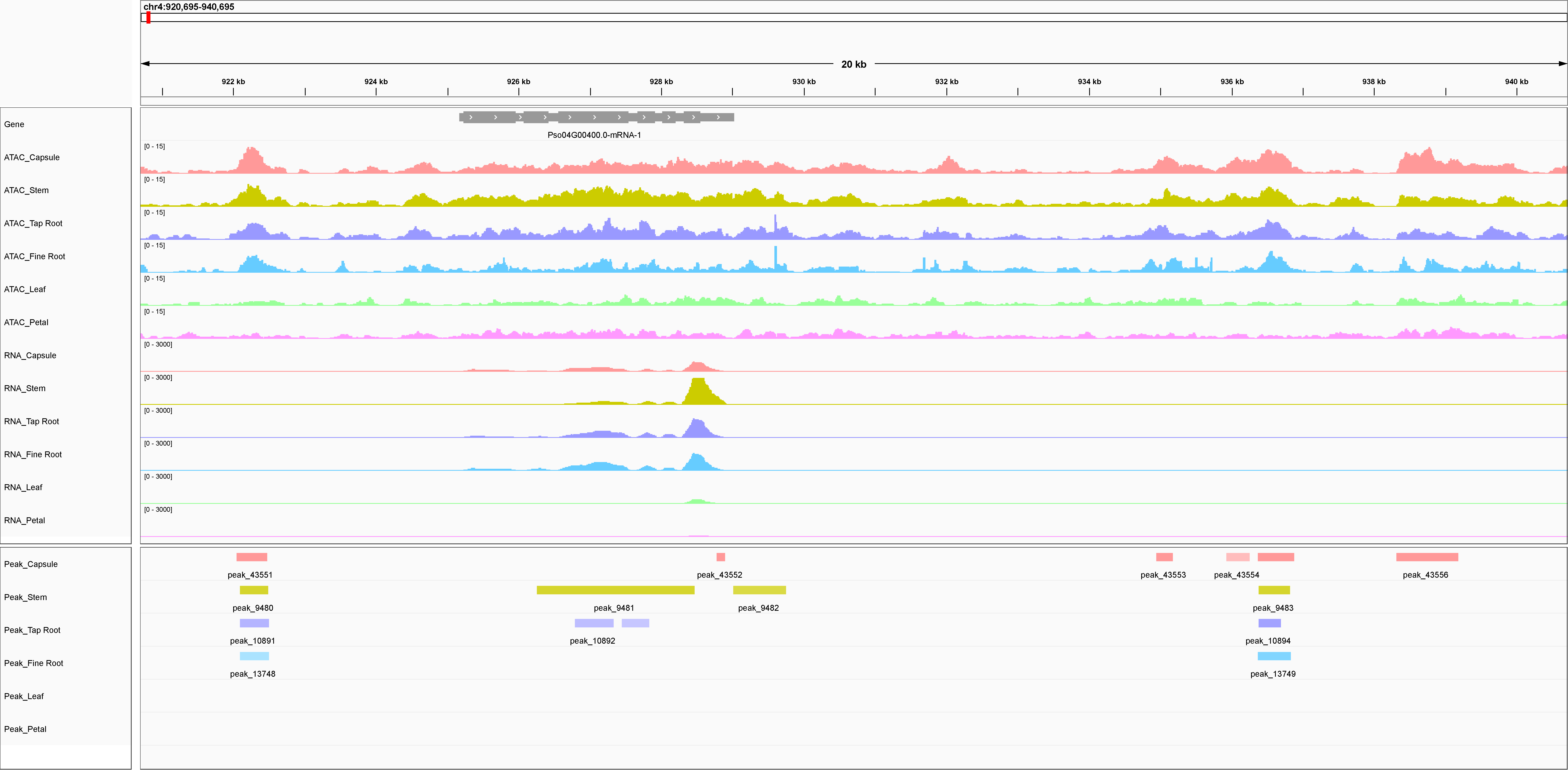

Supplement: Supplementary file 1 [file DataSheet1.ZIP › supplementary material/Supplementary Figure 5.tif]
